# Heterochromatin loss as a determinant of progerin‐induced DNA damage in Hutchinson–Gilford Progeria

**DOI:** 10.1111/acel.13108

**Published:** 2020-02-22

**Authors:** Alexandre Chojnowski, Peh Fern Ong, Mattheus Xing Rong Foo, David Liebl, Louis‐Peter Hor, Colin L. Stewart, Oliver Dreesen

**Affiliations:** ^1^ Developmental and Regenerative Biology Institute of Medical Biology Singapore Singapore; ^2^ Cell Ageing, Skin Research Institute Singapore Singapore Singapore; ^3^ A*STAR Microscopy Platform Singapore Singapore

**Keywords:** DNA damage, heterochromatin, HGPS, lamin A, progerin, senescence

## Abstract

Hutchinson–Gilford progeria is a premature aging syndrome caused by a truncated form of lamin A called progerin. Progerin expression results in a variety of cellular defects including heterochromatin loss, DNA damage, impaired proliferation and premature senescence. It remains unclear how these different progerin‐induced phenotypes are temporally and mechanistically linked. To address these questions, we use a doxycycline‐inducible system to restrict progerin expression to different stages of the cell cycle. We find that progerin expression leads to rapid and widespread loss of heterochromatin in G1‐arrested cells, without causing DNA damage. In contrast, progerin triggers DNA damage exclusively during late stages of DNA replication, when heterochromatin is normally replicated, and preferentially in cells that have lost heterochromatin. Importantly, removal of progerin from G1‐arrested cells restores heterochromatin levels and results in no permanent proliferative impediment. Taken together, these results delineate the chain of events that starts with progerin expression and ultimately results in premature senescence. Moreover, they provide a proof of principle that removal of progerin from quiescent cells restores heterochromatin levels and their proliferative capacity to normal levels.

## Graphical abstract

Hutchinson–Gilford progeria is an accelerated aging syndrome caused by a truncated mutant form of lamin A, called progerin. Progerin expression results in rapid and widespread heterochromatin loss in G1‐arrested cells, without causing any DNA damage. Removal of progerin from G1‐arrested cells restores heterochromatin levels and leaves no permanent growth defect. Importantly, progerin‐induced DNA damage occurs exclusively in late S‐phase and preferentially in cells with low heterochromatin.

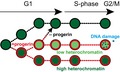

## 1 INTRODUCTION

Hutchinson–Gilford progeria (HGPS) is a sporadic, autosomal‐dominant premature aging syndrome, caused by a de novo point mutation in the *LMNA* gene (Dreesen & Stewart, 2011; Eriksson et al., 2003; Kubben & Misteli, 2017; De Sandre‐Giovannoli et al., 2003; Vidak & Foisner, 2016). HGPS patients exhibit early signs of premature aging, including alopecia and sclerotic skin, and die in their mid‐teens from cardiovascular complications. At the cellular level, fibroblasts derived from HGPS patients and normal cells expressing progerin display a broad spectrum of phenotypes, including nuclear abnormalities, loss of heterochromatin, DNA damage and premature senescence.

Previous studies reported that progerin expression leads to mitotic defects (Cao, Capell, Erdos, Djabali, & Collins, 2007; Dechat et al., 2007), whereas more recent findings suggested that both progerin and prelamin A may trigger DNA damage during DNA replication (Cobb, Murray, Warren, Liu, & Shanahan, 2016; Hilton et al., 2017; Wheaton et al., 2017). However, deciphering the causal and temporal links between the different progerin‐induced phenotypes remains challenging as the majority of studies have been conducted in patient‐derived cells, or cells constitutively expressing progerin, where immediate consequences of progerin expression and secondary effects arising from progerin‐induced senescence cannot be distinguished.

We previously reported a doxycycline‐inducible system to express physiological levels of progerin in isogenic primary‐ and TERT‐immortalized human dermal fibroblasts (NDF) and found that expression of TERT prevents progerin‐induced premature senescence (Chojnowski et al., 2015; Kudlow, Stanfel, Burtner, Johnston, & Kennedy, 2008). However, TERT did not prevent progerin‐induced heterochromatin loss and nuclear abnormalities (Chojnowski et al., 2015). This unique system allows us to distinguish what may be a cause or consequence of progerin‐induced senescence.

Here, we used this experimental system to temporally restrict progerin expression to particular cell cycle stages and to determine the consequences of transient progerin exposure. By inducing progerin expression in G1‐arrested cells, we demonstrate that progerin‐induced loss of peripheral heterochromatin does not require cells to undergo DNA replication or mitosis. In addition, progerin does not cause any DNA damage in G1‐arrested cells. We demonstrate that progerin‐induced DNA damage occurs exclusively during late stages of DNA replication when heterochromatin is normally replicated, prior to chromosome condensation and mitosis, and preferentially in cells with low levels of heterochromatin. Lastly, this inducible system allowed us to transiently express progerin in G1‐arrested cells and demonstrate that clearance of progerin in G1‐arrested cells restores heterochromatin levels without the need for DNA replication or mitosis and results in no proliferative impediment. Together, our results delineate the chain of events that occurs upon progerin expression across the cell cycle and ultimately results in cellular senescence. In addition, we demonstrate that some of the progerin‐induced defects can be reversed upon progerin removal without resulting in any lasting cell proliferation defects.

## 2 RESULTS

### 2.1 Progerin‐induced heterochromatin loss is independent of DNA replication and mitosis

We and others previously showed that progerin expression triggers extensive heterochromatin loss, a phenotype observed in both in vitro models and patient cells (Chojnowski et al., 2015; Scaffidi & Misteli, 2005; Shumaker et al., 2006). In addition, we demonstrated that TERT expression prevents progerin‐induced senescence, without alleviating heterochromatin loss, suggesting that the heterochromatin loss is not a consequence of cellular senescence (Chojnowski et al., 2015).

To further characterize the temporal dynamics of progerin‐induced heterochromatin loss and to investigate whether it is contingent upon DNA replication or mitosis, we restricted progerin expression to G1‐arrested cells and studied heterochromatin and progerin levels by quantitative single‐cell immunofluorescence microscopy.

**Figure 1:**
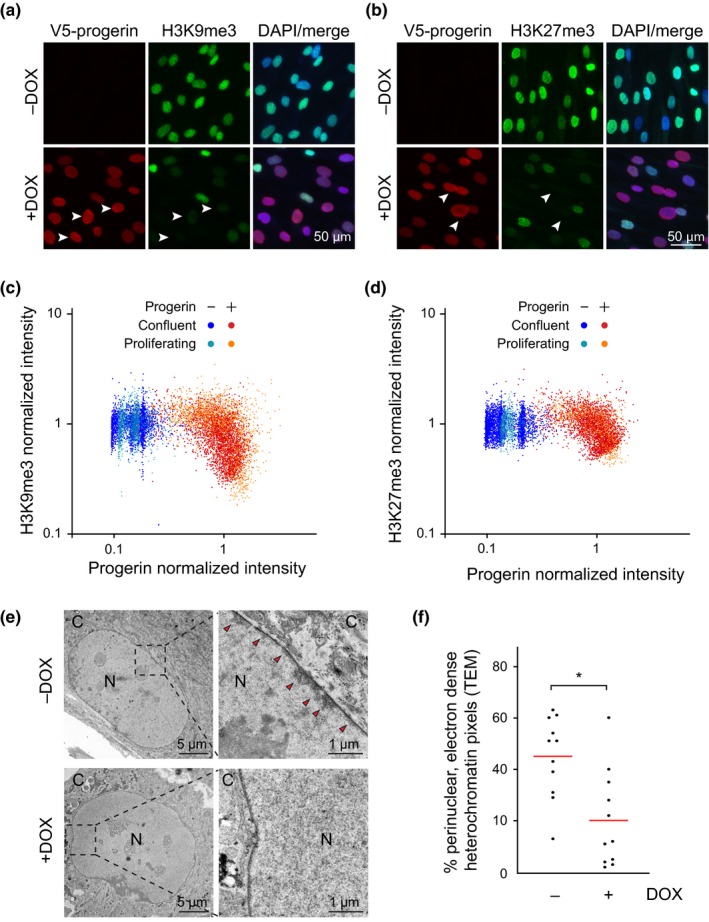
Progerin‐dependent loss of H3K9me3 and H3K27me3 heterochromatin marks in G1‐arrested confluent NDF. (a,b) Immunofluorescence microscopy of H3K9me3 (a) and H3K27me3 (b) staining in G1‐arrested cells in the presence or absence of progerin. V5‐progerin (V5 antibody) and H3K9me3/H3K27me3 antibodies are indicated, with DAPI overlay. Scale bar: 50 μm. Progerin‐expressing cells with extensive loss of H3K9me3 or H3K27me3 are marked by white arrowheads. (c,d) Scatter plot analysis of H3K9me3 (c) or H3K27me3 (d) and progerin levels in confluent or proliferating NDF in the presence (red, orange) or absence (blue, light blue) of progerin. H3K9me3 or H3K27me3 and progerin normalized intensities are plotted on Y and X axis, respectively. A total of ~9 × 10^3^ and ~7 × 10^3^ nuclei were quantified for H3K9me3 and H3K27me3 analysis, respectively, from 3 independent experiments. (e) Electron microscopy imaging of peripheral heterochromatin in confluent NDF in the presence (lower panel) or absence (upper panel, red arrowheads) of progerin. Nucleus (N) and cytoplasm (C) are indicated, scale bars: 5μm (left panels) and 1μm (right panels). (f) Quantification of perinuclear heterochromatin from TEM images in the absence (−DOX) and presence (+DOX) of progerin (see Figure S1‐1a) (*p < .05, *n* = 11 cells per condition, Mann‐Whitney test)

To achieve this, we grew cells to confluence, induced progerin expression and then quantified their heterochromatin levels. Upon induction of progerin, we observed a reduction of H3K9me3 and H3K27me3 heterochromatin marks (Figure 1a–d) and of heterochromatin levels (Figure 1e,f, Figure S1‐1a & Figure S1‐2a,b). Significantly, the correlation between progerin expression and H3K9me3 and H3K27me3 loss was similar between G1‐arrested and control cells allowed to proliferate (Pearson *r* = −0.44/−0.42 for H3K9me3 and −0.40/−0.56 for H3K27me3 between confluent and proliferating cells, respectively). We then used 3D structured illumination microscopy (3D‐SIM) analysis to determine the spatial distribution of H3K27me3 and found that progerin‐induced loss of heterochromatin was not due to re‐localization of peripheral heterochromatin towards the centre of the nucleus (Figure S1‐1b,c). These results demonstrate that progerin expression triggers rapid and extensive loss of peripheral heterochromatin in both quiescent and proliferating cells, without the need for DNA replication or mitosis, suggesting that the mechanism of the heterochromatin loss in proliferative and nonproliferative cells may be similar.

### 2.2 Progerin‐induced DNA damage occurs exclusively in replicating cells

**Figure 2:**
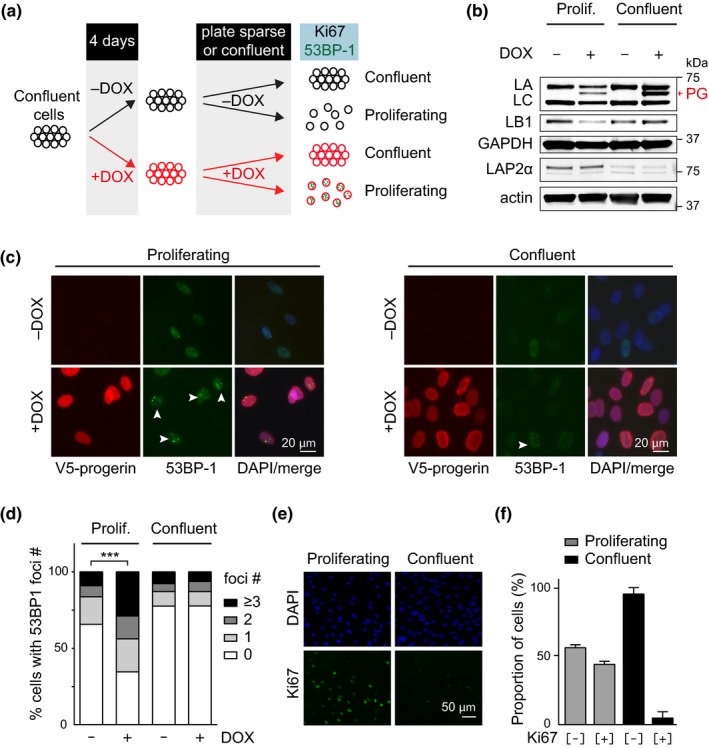
Progerin‐induced DNA damage is restricted to proliferating cells. (a) Schematic representation of the experimental set up. (b) Western blotting showing doxycycline‐dependent progerin expression in proliferating and confluent NDF. Progerin migrates between lamin A and C as indicated (red arrowhead). Lamin A (LA), lamin C (LC), progerin (PG), lamin B1 (LB1), LAP2α, GAPDH and actin are indicated. (c) Immunofluorescence microscopy showing progerin‐induced 53BP‐1 foci (white arrowheads) in proliferating (left panel) or confluent cells (right panel). V5‐progerin (V5 antibody) and 53BP‐1 foci (53BP‐1 antibody) are indicated. Scale bar: 20 μm. (d) Quantification of DNA damage foci (0, 1, 2, 3 or more 53BP‐1 foci), in proliferating or confluent cells in the absence or presence of progerin (***p < .001, *n* = 3, χ^2^ test). (e) Immunofluorescence microscopy showing Ki‐67 staining in proliferating (left panels) and confluent cells (right panels). DAPI and Ki‐67 antibody are shown on top and bottom panels, respectively. Scale bar: 50 μm. (f) Quantification of the percentage of Ki‐67‐positive and Ki‐67‐negative cells in proliferating or confluent cultures (grey and black bars, respectively)

Next, we tested whether the progerin‐induced heterochromatin loss and DNA damage could be temporally separated to different stages of the cell cycle. Previous studies showed that progerin‐induced DNA damage can occur during DNA replication, although significant amounts of DNA damage were also apparent in G1 cells (Wheaton et al., 2017). To precisely determine the timing of progerin‐induced DNA damage, we temporally restricted progerin expression to G1‐arrested cells using our doxycycline‐inducible lentiviral system. We grew NDF harbouring doxycycline‐inducible progerin to complete confluence and induced progerin expression by adding doxycycline to the cell culture media, while control cells remained uninduced (Figure 2a). After 4 days, cells were re‐plated either at confluency (to remain G1‐arrested) or sparsely (to re‐initiate proliferation). Progerin expression levels were monitored by Western blotting and immunofluorescence microscopy (Figure 2b,c). As previously described, G1‐arrested cells expressed low levels of LAP2α (Figure 2b) and expectedly did not express the proliferation marker Ki‐67 (Figure 2e,f) (Dreesen et al., 2013; Naetar et al., 2008; Pekovic et al., 2007). Progerin‐expressing cells allowed to proliferate exhibited a significant increase in DNA damage and senescence‐associated loss of lamin B1, while DNA damage and lamin B1 levels remained unchanged in confluent progerin‐expressing cells, despite expressing higher progerin levels (Figure 2b–d, Figure S2a).

We then independently confirmed that proliferation was necessary to allow for progerin‐induced DNA damage by growing NDF to confluence within silicon scaffolds and induced progerin expression by addition of doxycycline (Figure S2b). After 4 days of induction, the scaffolds were removed allowing cells at the edge of the cultures to migrate and proliferate, while contact‐inhibited cells at the centre remained G1‐arrested. Progerin expression and DNA damage accumulation were assessed after an additional 3 days, and a significant increase in DNA damage was observed exclusively in progerin‐expressing cells at the proliferating edges of the cultures. In contrast, cells within the centre did not exhibit any increased DNA damage (Figure S2c,d).

Taken together, these results demonstrate that progerin‐induced DNA damage occurs exclusively in replicating cells, despite G1‐arrested cells retaining normal DNA damage response capabilities. This is in stark contrast to the heterochromatin loss induced by progerin expression, which also affects G1‐arrested cells.

### 2.3 Progerin‐induced DNA damage occurs preferentially in cells with low heterochromatin and specifically during late stages of DNA replication, prior to chromosome condensation

**Figure 3:**
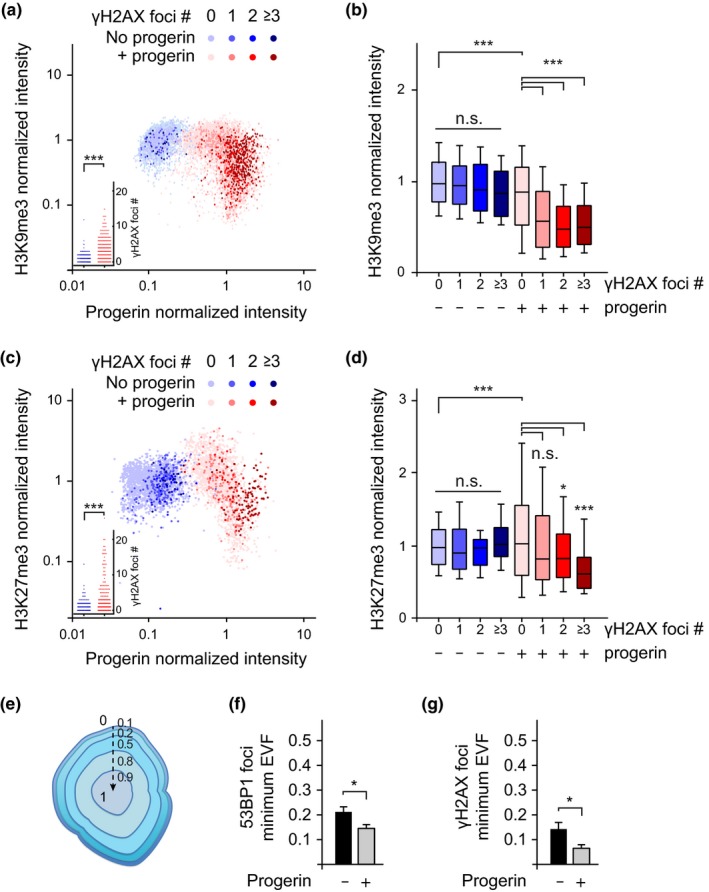
Preferential accumulation of DNA damage foci in cells with lower levels of H3K9me3 and H3K27me3. (a) Scatter plot analysis of H3K9me3, progerin expression and γH2AX DNA damage foci number per nucleus, in NDF expressing progerin (red) or nonexpressing controls (blue). For each nuclei, H3K9me3 and progerin normalized intensities are plotted on Y and X axis, respectively, while the number of DNA damage foci is represented by colour intensity (light to dark colour: 0, 1, 2, 3 or more DNA damage foci per nucleus). Inset: DNA damage counts of the same data (***p < .001, Student's *t* test). A total of ~ 1x10^4^ cells from 3 independent experiments were analysed. (b) Box plot of data shown in (a), whiskers represent 10–90 percentile (***p < .001, one‐way ANOVA with Tukey's post‐test). (c) Scatter plot analysis of H3K27me3, progerin expression and γH2AX DNA damage foci number. Inset: DNA damage counts of the same data (***p < .001, Student's *t* test). A total of ~ 4x10^3^ cells from 3 independent experiments were analysed. (d) Box plot of the same data, whiskers represent 10–90 percentile (***p < .001, *p < .05, one‐way ANOVA with Tukey's post‐test). (e,f) 3D‐SIM analysis of DNA damage foci. Illustration of the Eroded Volume Fraction (EVF) index to assess proximity to the nuclear lamina, from closest (0) to furthest (1) (reproduction of Figure S1‐1b). (f,g) Quantification of the average minimum EVF value for 53BP‐1 (f) and γH2AX (g) DNA damage foci, ± progerin. A total of 225 53BP‐1 and 135 γH2AX DNA damage foci were analysed. (*p < .05, Student's *t* test)

Heterochromatin decompaction renders some cell types more susceptible to DNA damage (Di Micco et al., 2011). To test whether progerin‐expressing cells with low levels of heterochromatin were more prone to accumulate DNA damage, we quantified heterochromatin levels (H3K27me3 and H3K9me3), DNA damage foci (by γH2AX) and progerin levels (by v5‐tag) in proliferating dermal fibroblasts (NDF) in the presence or absence of progerin (Figure 3). We observed an inverse correlation between progerin‐induced DNA damage and heterochromatin levels: Cells with increased numbers of DNA damage foci (>1 for H3K9me3 and >2 for H3K27me3) had significantly lower levels of H3K9me3 or H3K27me3, respectively (Figure 3a,c; dark red data points and 3b, 3d graphs). This correlation was not observed in control cells (Figure 3a,c; blue data points and 3b, 3d blue box plots). These results demonstrate that cells with low levels of heterochromatin are more susceptible to progerin‐induced DNA damage.

We then used 3D‐SIM imaging to determine the spatial localization of progerin‐induced DNA damage. The radial position of foci within each nucleus was quantified using the Eroded Volume Fraction as normalized index (EVF, Figure 3e) (Ballester et al., 2008). This analysis revealed that DNA damage foci, visualized by 53BP‐1 and γH2AX, tend to localize closer to the nuclear lamina in the presence of progerin (Figure 3f,g).

**Figure 4:**
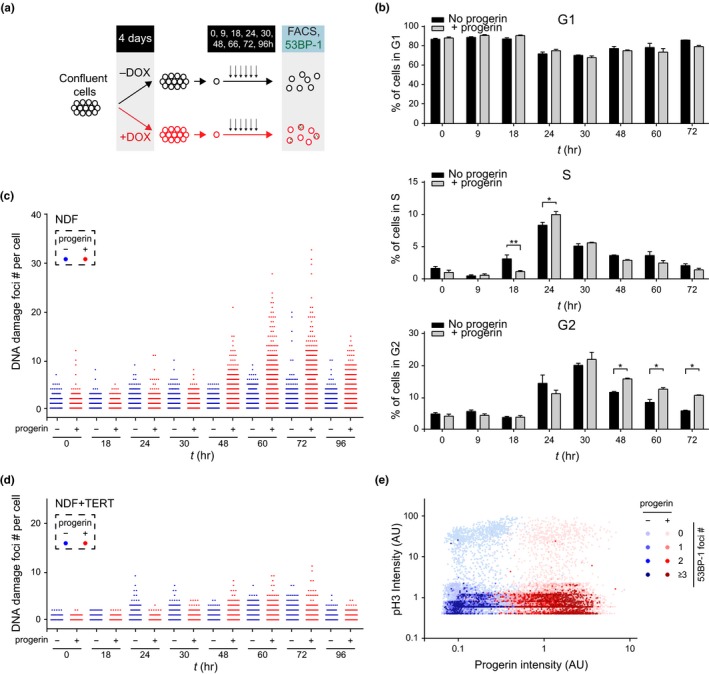
Progerin‐induced DNA damage occurs in late S‐phase and is associated with a persistent G2 arrest. (a) Schematic representation of the experimental design. (b) Cell cycle profile of control (black bars) and progerin‐expressing (grey bars) NDF at each time point (in hours) after release from G1‐arrest by FACS (% of cells in G1, S‐phase and G2 are shown in top, middle and lower panels, respectively; *p < .05, **p < .01, *n* = 3, two‐way ANOVA with Sidak's post‐test). (c) DNA damage accumulation in control (blue) and progerin‐expressing (red) NDF upon release from G1. The number of 53BP‐1 foci per cell is displayed at each time point. A total of ~1 × 10^5^ cells from 3 independent experiments were analysed. (d) Quantification of DNA damage foci in control (blue) and progerin‐expressing (red) TERT‐positive NDF upon G1 release. A total of 7 × 10^4^ cells from 2 independent experiments were analysed. (e) Scatter plot analysis of phospho‐H3 (pH3), progerin expression and 53BP‐1 DNA damage foci per nucleus, in control (blue) and progerin‐expressing (red) NDF upon release from G1. Data from all time points post‐G1‐arrest release (0–96 hr) are represented. Phospho‐H3 and progerin normalized intensities are plotted on Y and X axis, respectively, while the number of DNA damage foci is represented by colour intensity. From light to dark colour: 0, 1, 2, 3 or more DNA damage foci. ~9 × 10^4^ cells from 2 independent experiments were analysed

Progerin‐induced DNA damage necessitates cell proliferation (Hilton et al., 2017; Wheaton et al., 2017), arises in cells with lower levels of heterochromatin and is prevented by TERT. Since fragile sites like telomeres at the nuclear periphery and heterochromatin are all replicated late in S‐phase (Arnoult et al., 2010; Rhind & Gilbert, 2013), we asked whether the onset of DNA damage could coincide with the timing of normal heterochromatin replication in late S‐phase. To address this question, we grew NDF harbouring doxycycline‐inducible v5‐progerin to confluence, thereby synchronizing them in G1. We then released cells from G1‐arrest in the presence or absence of progerin by plating them sparsely and monitored their cell cycle progression by FACS. We concomitantly assessed the dynamics of DNA damage accumulation by quantifying 53BP‐1 and γH2AX foci at different time points after release (Figure 4a–d, Figure S4‐1a–d). Progerin expression levels were comparable between NDF and TERT‐expressing NDF, as expected (Figure S4‐1a,b). Cells underwent DNA replication and reached G2 ~24–30 hr after release from G1‐arrest, with no obvious delay between progerin‐expressing and control cells. However, a significantly higher proportion of the progerin‐expressing cells remained in G2 from 48 hr throughout the duration of the experiment. Importantly, the elevated number of cells in G2 coincided with the accumulation of progerin‐induced DNA damage, as shown by quantification of 53BP‐1 and γH2AX foci (Figure 4c and Figure S4‐1d). In addition, DNA damage foci numbers remained at background level throughout the first 30 hr of the experiment, indicating that DNA replication is required for the appearance of progerin‐induced DNA damage (Figure 4c, Figure S4‐1d). Both the increased number of cells in G2 and the elevated levels of DNA damage persisted until the end of the time course (Figure 4b,c, Figure S4‐1d) and were rescued by expression of TERT (Figure 4d, Figure S4‐1c).

To assess whether progerin expression per se hampers the recruitment of DNA damage response factors to damaged DNA, we determined the temporal dynamics of 53BP‐1 and γH2AX foci formation upon treatment with the chemotherapeutic agent doxorubicin. As shown in Figure S4‐2a,b, we did not detect any significant delay in the DNA damage response in progerin‐expressing cells (Figure S4‐2a,b).

These observations suggested that progerin‐dependent DNA damage occurs either late during DNA replication (S) or at the onset of chromosome condensation and mitosis (late G2/ early M). To distinguish between these two possibilities, we first co‐stained cells with phospho‐histone 3 (p‐H3), a marker for chromosome condensation and mitosis (Hans & Dimitrov, 2001), progerin (v5) and DNA damage markers 53BP‐1 or γH2AX, at different time points during the experiment (0, 18, 24, 30, 48, 60, 72, 96 hr). Among the ~1.2 × 10^5^ cells analysed, the accumulation of 53BP‐1 or γH2AX foci was restricted to p‐H3‐negative cells, indicating that DNA damage occurred prior to chromosome condensation and mitosis (Figure 4e and Figure S4‐1e). Secondly, we introduced mCherry‐BP1‐2 (mCherry fused to amino acids 1220–1711 of 53BP‐1) (Dimitrova, Chen, Spector, & Lange, 2008) into progerin‐expressing cells and used time‐lapse microscopy to visualize sites of progerin‐induced DNA damage foci in real time. In agreement with our time course results, cells with multiple DNA damage foci appeared between 30–60 hr after release from G1, prior to mitosis. DNA damage‐positive cells displayed hallmarks of senescence, including enlarged nuclei and a flattened morphology and did no longer divide (Figure 4, Movie S1).

Together, these results indicate that TERT‐preventable progerin‐induced DNA damage occurs exclusively during late stages of DNA replication, when heterochromatin is normally replicated, prior to chromosome condensation and mitosis.

### 2.4 Removal of progerin in G1‐arrested cells restores heterochromatin levels and results in no permanent proliferation defect

**Figure 5:**
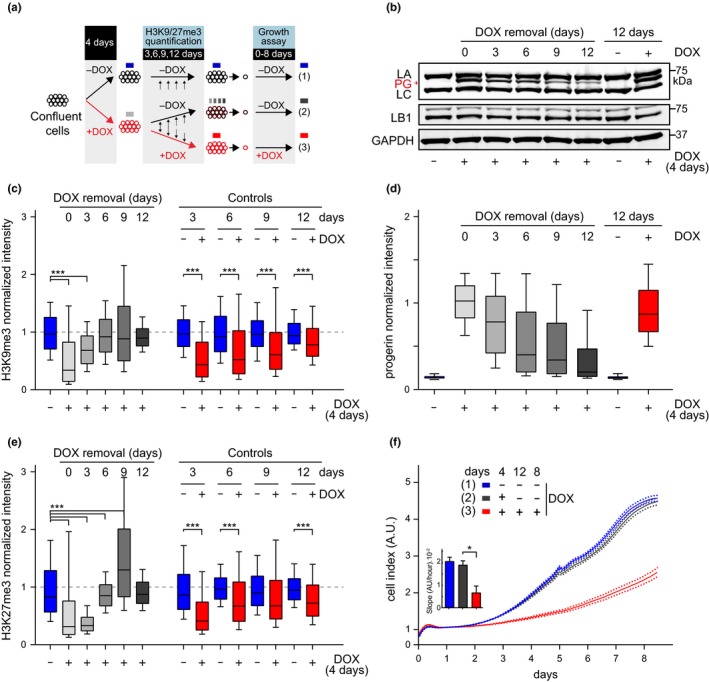
Restored heterochromatin levels and cell proliferation upon progerin removal in G1‐arrested NDF. (a) NDF were grown to confluence and exposed to progerin expression (+DOX, red circles) for 4 days. Nonexposed cells (−DOX, black circles, blue box) served as a control. After 4 days, progerin expression was either turned off (−DOX; black/red circles, grey box) or left on (+DOX, red circles, red box) and kept confluent for an additional 12 days. Proliferation rates were compared for cells never exposed to progerin (blue), cells in which progerin was removed (grey) and cells constitutively exposed to progerin (red). (b) Western blotting showing doxycycline‐dependent progerin expression and removal in confluent primary fibroblasts. Progerin (PG), lamin A (LA) and lamin C (LC), lamin B1 (LB1) and GAPDH are indicated. Progerin intensities quantified by single‐cell immunofluorescence microscopy at each day are indicated in panel (d). A total of ~ 7.7 × 10^4^ nuclei were quantified for both H3K9me3 and H3K27me3 quantifications, from 2 independent experiments. Whiskers represent 10–90 percentile. (c,e) Box plot representation of H3K9me3 (c) and H3K27me3 (e) levels at the indicated times post doxycycline removal (0, 3, 6, 9, 12 days; grey bars) or in controls never (blue) or constitutively (red) exposed to progerin. A total of ~3.8 × 10^4^ and ~3.9 × 10^4^ nuclei were quantified for H3K9me3 and H3K27me3, respectively, from 2 independent experiments, ***p < .001, one‐way ANOVA with Sidak's post‐test. Whiskers represent 10–90 percentile. (d) Box plot representation of progerin normalized intensity per nucleus, in NDF induced to express progerin (grey, red) or noninduced (blue/black). (f) Growth curve of control and primary fibroblasts continuously expressing progerin (red line), not expressing progerin (blue line), or expressing progerin for 4 days while confluent and without induction thereafter (black). Dotted lines indicate *SEM* (*n* = 3). Inset: growth rate after 8 days, error bars indicate *SEM* (*p < .05, one‐way ANOVA with Tukey's post‐test)

Our results demonstrate that progerin‐induced heterochromatin loss is a dynamic process occurring independently of DNA replication. However, it remained unclear whether the heterochromatin loss was a permanently acquired phenotype of cells expressing progerin or whether it could be restored upon progerin removal. To address this question, we grew cells to confluence, induced progerin expression for 4 days and subsequently withdrew doxycycline from the cell culture media. After doxycycline removal, we monitored progerin and heterochromatin levels at 3, 6, 9 and 12 days (Figure 5a). Cells expressing progerin throughout the experiment and noninduced cells served as controls. As shown in Figure 5b,d, progerin levels gradually declined upon removal of doxycycline. Importantly, H3K9me3 and H3K27me3 levels were restored concurrently with declining progerin levels (Figure 5c,e). In contrast, cells that were permanently exposed to progerin exhibited low levels of heterochromatin, while uninduced cells showed normal levels. These results demonstrate that (a) heterochromatin levels rapidly increase upon progerin removal and (b) that this process does not require DNA replication or mitosis.

To investigate whether cells transiently exposed to progerin expression exhibit any long‐term defects, we expressed progerin in G1‐arrested cells for 4 days, upon which doxycycline was removed (Figure 5a). 8 days after doxycycline removal, we plated cells at low density to allow proliferation and assessed their proliferative capacity. Strikingly, the proliferation rate, DNA damage levels and senescence‐associated‐β‐galactosidase (SA‐β‐gal) staining of cells transiently exposed to progerin were indistinguishable from cells that were never exposed to progerin (Figure 5f, Figure S5a–d). In contrast, cells constitutively exposed to progerin exhibited impaired proliferation, increased DNA damage and stained positive for SA‐β‐gal as described previously (Benson, Lee, Aaronson, & a, 2010; Chojnowski et al., 2015; Kudlow et al., 2008). These results demonstrate that removal of progerin from cells prior to DNA replication restores heterochromatin levels without resulting in any lasting proliferative defect. In addition, these results highlight that chromatin compaction upon progerin removal does not require the cell to undergo DNA replication or mitosis.

## 3 DISCUSSION

The loss and disorganization of heterochromatin during aging is now well‐documented, from the loss of lamina‐associated heterochromatin during normal and premature aging (McCord et al., 2013; Shumaker et al., 2006; Tsurumi & Li, 2012; Whitton et al., 2018; Zhang et al., 2015), to oxidative‐stress induced aging (Osanai et al., 2018) and aging of the immune system (Keenan & Allan 2018). Heterochromatin loss is accompanied by extensive remodelling of the nuclear lamina (Chojnowski, Ong, & Dreesen, 2014; Dreesen et al., 2013), and it has been proposed to be determinant of aging in yeast, flies and worms (Tsurumi & Li, 2012). However, the functional role of the heterochromatin loss in HGPS and how it relates to other progerin‐induced defects remains unclear. By temporally restricting the expression of progerin, we demonstrate for the first time that progerin‐dependent heterochromatin loss is a rapid and dynamic process, independent of DNA replication or mitosis. Furthermore, we show that this loss can be reversed, as progerin removal restored heterochromatin levels and resulted in no permanent damage or proliferation defect. These results are reminiscent of the reprogramming of HGPS fibroblasts into induced pluripotent stem cells, during which expression of A‐type lamins and progerin is silenced and heterochromatin levels are restored (Chen et al., 2017; Zhang et al., 2011).

What is the physiological relevance of the heterochromatin loss in progeric cells? We demonstrate for the first time that progerin‐induced DNA damage occurs preferentially in cells with lower levels of heterochromatin. This is in agreement with previous results showing that the relaxation of heterochromatin results in increased DNA damage, a property used by HDAC inhibitors for cancer treatment (Di Micco et al., 2011). In addition, we find that progerin expression per se does not alter the temporal dynamics of the DNA damage response. However, since we analysed the early consequences of progerin expression, we cannot exclude the possibility that cells with an extended history of progerin exposure may display such impediment (Liu et al., 2005).

Importantly, we demonstrate that heterochromatin loss by itself is not sufficient for progerin to induce DNA damage: progerin‐expressing cells also have to undergo DNA replication to accumulate DNA damage, as suggested earlier (Wheaton et al., 2017). Moreover, we provide evidence that the onset of progerin‐induced DNA damage occurs exclusively during the late stages of DNA replication, prior to chromosome condensation and mitosis. Interestingly, this corresponds with the replication timing of heterochromatin and telomeres near the periphery, late in S‐phase (Arnoult et al., 2010; Rhind & Gilbert, 2013). The timing of the progerin‐induced DNA damage is also consistent with the fact that TERT’s ability to rescue such DNA damage (Benson et al., 2010; Chojnowski et al., 2015; Kudlow et al., 2008; Li et al., 2019) is only apparent during DNA replication and leaves background DNA damage foci occurring at other stages entirely unaffected. However, the precise mechanisms how TERT, or hTERT mRNA prevent, or repair progerin‐induced DNA damage, remains to be determined.

The mechanism by which progerin triggers such rapid and widespread heterochromatin loss remains elusive. Previous studies showed decreased levels of several chromatin‐modifying enzymes in progeric cells such as HP1α, Suv39 and EZH2 (McCord et al., 2013; Scaffidi & Misteli, 2005; Shumaker et al., 2006). However, since these experiments were done mostly in HGPS cells, it remains difficult to ascertain whether these changes were direct consequences of progerin expression, preceding senescence or DNA damage‐related phenotypes.

Lastly, not all cells with low levels of heterochromatin accumulate progerin‐induced DNA damage during DNA replication. Stochastic processes may account for this variability, but additional factors could also come into play. Hilton and colleagues suggested that progerin or prelamin A sequester the proliferating cell nuclear antigen (PCNA) away from the replication fork, leading to fork collapse and subsequent DNA damage signalling activation (Cobb et al., 2016; Hilton et al., 2017).

Together, we demonstrate that progerin expression in G1‐arrested cells triggers rapid and extensive heterochromatin loss without causing any DNA damage. Progerin‐induced DNA damage accumulates exclusively during late stages of DNA replication, prior to chromosome condensation, and preferentially in cells with low levels of heterochromatin. The necessity for cell proliferation may be more central to the pathophysiology of progeria than expected: studies have shown that multiple cell types undergo proliferation in tissues previously deemed postmitotic. For HGPS patients, whose cardiovascular system is particularly affected, this includes two of the heart's fundamental components: vascular smooth muscle cells and cardiomyocytes (Anversa & Nadal‐Ginard, 2002; Bergmann et al., 2009, 2015). In addition, the importance of cycling cell types in progeria is underscored by a recent report showing that progerin expression restricted to vascular smooth muscle cells recapitulates many of the HGPS‐associated cardiovascular defects (Hamczyk, Campo, & Andrés, 2018). Lastly, we provide evidence that heterochromatin levels are rapidly restored upon progerin removal and that transient exposure to progerin in G1‐arrested cells does not result in any lasting proliferative defect. These results define the causal and temporal link between progerin‐induced heterochromatin loss, DNA replication and DNA damage. They delineate a chain of events that commences with progerin expression and ultimately results in premature senescence, an essential step towards a better understanding of the pathophysiology of progeria.

## 4 EXPERIMENTAL PROCEDURES

### 4.1 Cell culture & western blotting

NDF culture, progerin expression and Western blotting were conducted as described previously (Chojnowski et al., 2015).

### 4.2 Culture of cells in silicone inserts

Ibidi cell culture inserts (Ibidi Cat. # 80209) were mounted on 24‐mm‐diameter coverslips (Nunc Cell Culture, Thermo Fisher). NDF harbouring doxycycline‐inducible pTRIPZ‐v5‐progerin were grown to confluence within culture inserts. NDF were induced ± 1 μg/ml doxycycline for 3 days and culture inserts removed to initiate cell proliferation and migration at the edge. Cells were fixed 72 hr postinsert removal and analysed by immunofluorescence microscopy.

### 4.3 Doxorubicin treatment

NDF + TERT harbouring doxycycline‐inducible pTRIPZ‐v5‐progerin were grown to confluence on chambered cover glass (Nunc). Progerin expression was induced for 4 days. Cells were treated with 250 ηM doxorubicin for 30min and fixed after 0.5, 1.5, 2.5, 4, 6, 8 and 10 hr. DNA damage foci were visualized by 53BP‐1 and γH2AX staining.

### 4.4 Immunofluorescence microscopy

Cells were seeded onto chambered cover glass (Nunc) and fixed. Immunofluorescence staining was conducted as described previously (Chojnowski et al., 2015). Images were acquired using Olympus FV1000 inverted laser scanning confocal microscope, IX‐83 inverted wide‐field fluorescent microscope (Olympus), Ts2‐FL inverted microscope (Nikon) or DeltaVision OMX Blaze microscope (Olympus) (Chojnowski et al., 2015). For time lapse, images were taken every 30min on the Olympus IX‐83 fluorescent microscope, for the duration of the experiment. All images were processed and quantified using ImageJ (Schindelin et al., 2012) and CellProfiler (Carpenter et al., 2006). Nuclei were segmented using the Otsu thresholding method, and for each nuclei, average intensities were measured, unless otherwise stated for EVF analysis. EVF analysis was performed using TANGO (Ollion et al., 2013) as an implementation of the EVF described previously (Ballester et al., 2008).

### 4.5 Transmission electron microscopy

Cells were grown on glass coverslips and processed by flat‐embedding for electron microscopy as follows: samples were fixed in 2.5% glutaraldehyde and 4% formaldehyde in 0.1M Sodium Cacodylate Buffer (pH7.4) followed by postfixation in 1% osmium tetroxide. After washing in distilled water, samples were dehydrated in ethanol (including an overnight staining with 1% uranyl acetate) and finally in propylene oxide. Dehydrated samples were embedded in epoxy resin EPON 812 (Serva) and polymerized at 60ºC for 48 hr. Horizontal ultrathin sections were cut with diamond knife (Diatome) on EM UC7 ultramicrotome (LEICA Microsystems). Sections were collected on formvar–carbon‐coated copper grids (EMS), postcontrasted with lead citrate and analysed under a JEM1010 transmission electron microscope (JEOL) operating at 80 kV. Images were acquired with SIA model 12C CCD camera (16bit, 4K). Perinuclear heterochromatin levels were quantified from TEM images using ImageJ, as the proportion of dark pixels along the nuclear lamina. Pixels were considered as dark (electron‐dense) when their intensity was below the mean grey value of typical heterochromatin clusters.

### 4.6 Cell cycle analyses by FACS

Cells were trypsinized at indicated time points, using 0.25% Trypsin/EDTA (GIBCO, Thermo Scientific), and trypsin was neutralized in cell culture media containing 15% FBS. Cells were spun at 300× *g* for 5min, resuspended in 10% FBS in PBS and fixed by adding ice‐cold 100% ethanol, drop‐wise, while shaking the tube. Cells were then kept at −20ºC for at least 4 hr or until they are ready for staining. DNA was stained using 10 µg/ml propidium iodide in PBS with 10 µg/ml RNase A, for 1 hr at room temperature, in the dark. Samples were analysed on the BD^TM^ LSRII (Becton Dickinson) flow cytometer.

### 4.7 Antibodies

Primary antibodies used in this study are as follows: 53BP‐1 (Novus Biologicals, NB100‐304), γH2AX (Millipore; 05–636), H3K9me3 (abcam; ab8898), H3K27me3 (Millipore; 07–449), v5 (abcam; ab9137), phospho‐Histone H3 (ser 10) (Cell Signaling; #9706), LAP2α (abcam; ab5162), Lamin A/C (Millipore; MAB3211), Lamin B1 (YenZym), Ki67 (abcam; ab16667), Actin (Sigma, A5441), GAPDH (Sigma, G9545).

### 4.8 Senescence‐associated‐β‐galactosidase staining

Senescence‐associated‐β‐galactosidase staining was conducted as described (Chojnowski et al., 2015; Dimri et al., 1995). Images were acquired using a 10x objective on the Nikon Ts2‐FL inverted. ImageJ was used to quantify SA‐β‐galactosidase‐positive cells.

### 4.9 Statistical analysis

Data and statistical analyses were performed using Excel and GraphPad Prism software. Results are shown as mean ± SEM/*SD*, and box plot whiskers indicate 10–90 percentile, unless otherwise stated. Data were analysed using one‐ or two‐way ANOVA and Tukey's post hoc test if required, as well as chi‐squared, two‐tailed Student's *t* test and Pearson correlation coefficients, as appropriate. *p*‐values below.05 were considered significant. For experiments comprising large sample sizes, significant *p*‐values were reported only when the effect size was at least considered "small" (effect size above 0.2), as described (Chavalarias, Wallach, Li, & Ioannidis, 2016; Sullivan & Feinn, 2012).

## CONFLICT OF INTEREST

Authors declare that there is no conflict of interest.

## AUTHOR CONTRIBUTIONS

OD designed experiments and wrote the manuscript with AC. AC, PFO, MXRF, DL, LPH and OD performed experiments and analysed the data. CLS reviewed the manuscript.

## Data Availability

The data that support the findings of this study are available from the corresponding author upon reasonable request.
