# Supplementary figures and images for: Heterochromatin loss as a determinant of progerin‐induced DNA damage in Hutchinson–Gilford Progeria
Source: Aging Cell. 2020 Feb 22;19(3):e13108. doi: 10.1111/acel.13108 (PMC7059134; doi:10.1111/acel.13108)

A

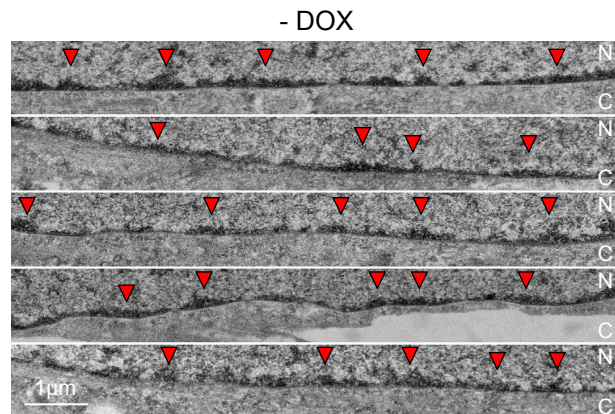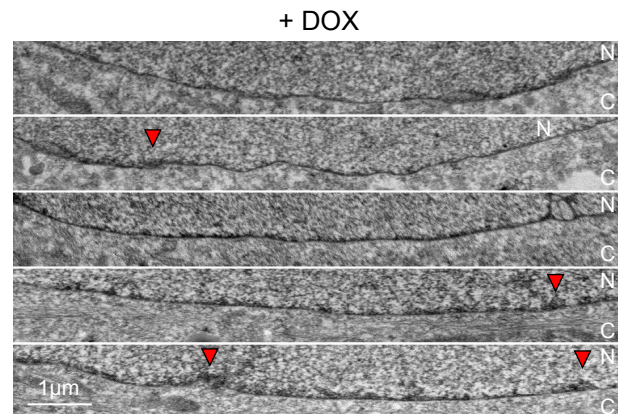

B

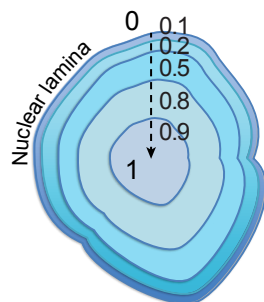

C

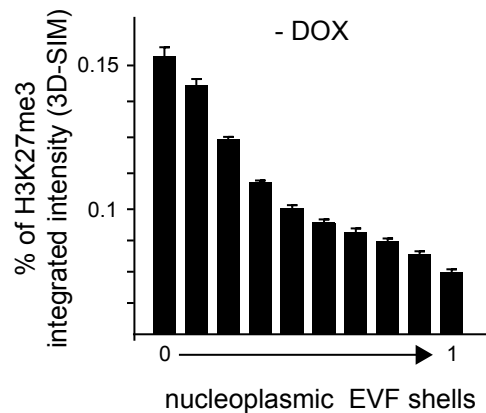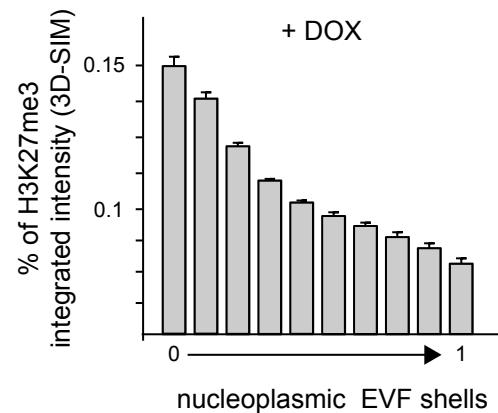

Supplement: Supplementary file 1 [file ACEL-19-e13108-s001.pdf]

A

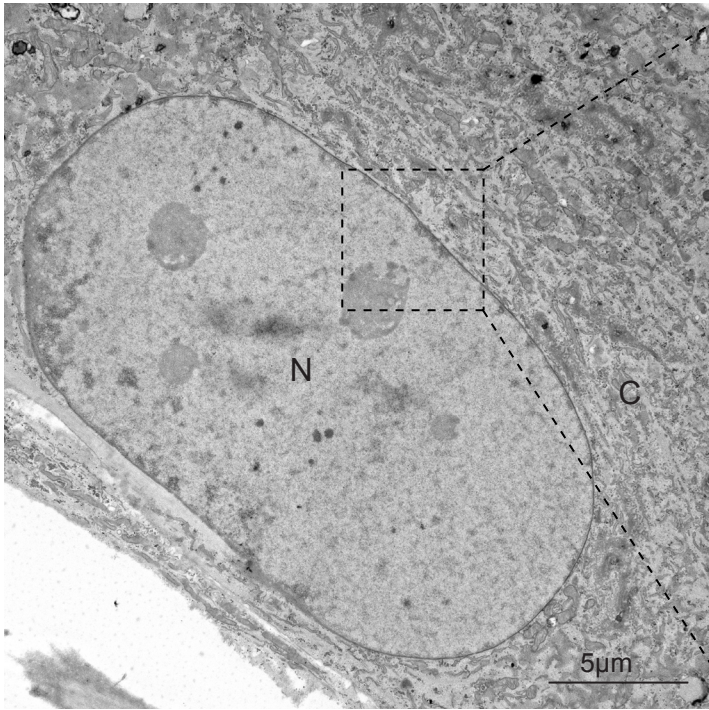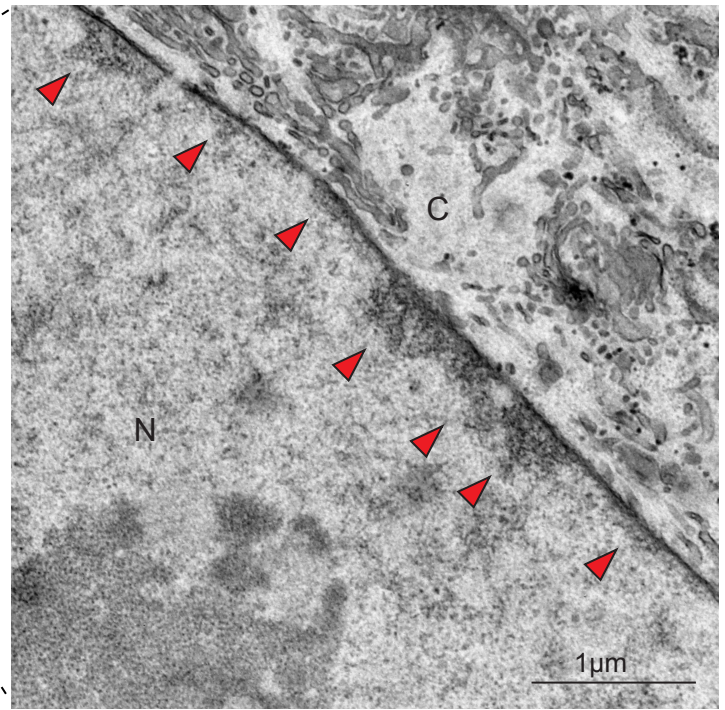

B

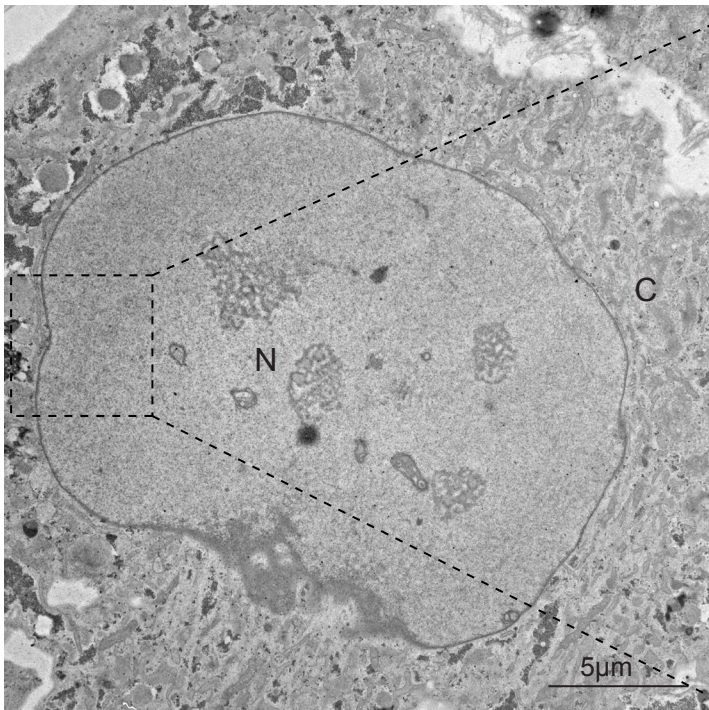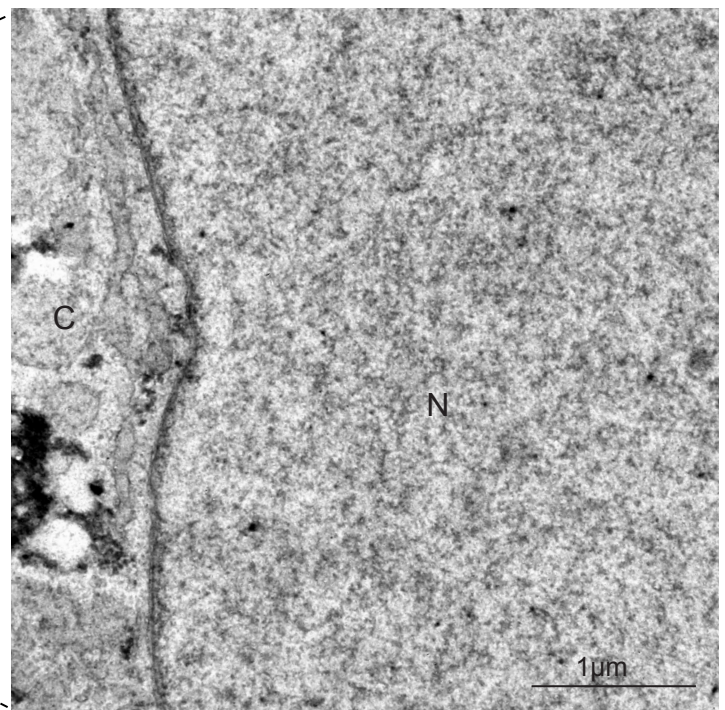

Supplement: Supplementary file 2 [file ACEL-19-e13108-s002.pdf]

A

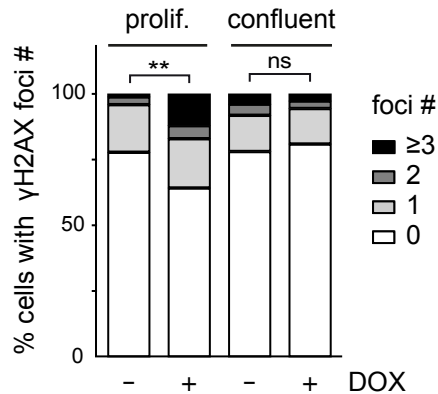

B

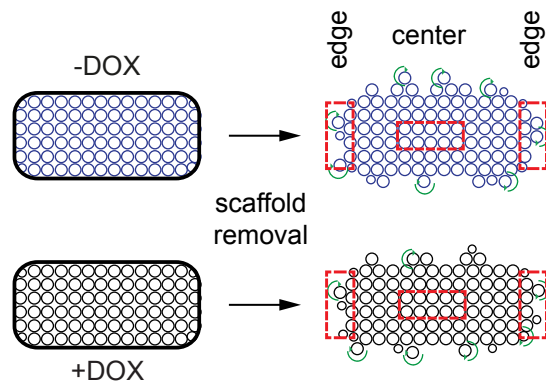

C

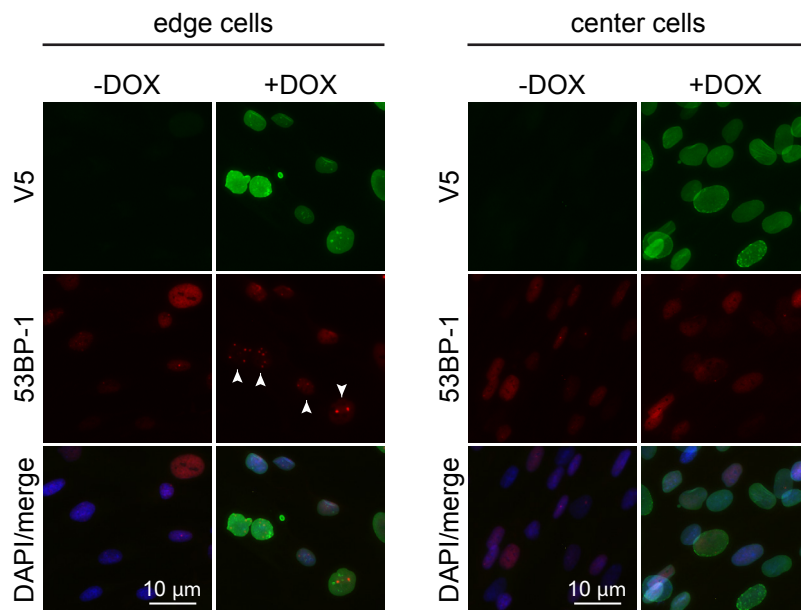

D

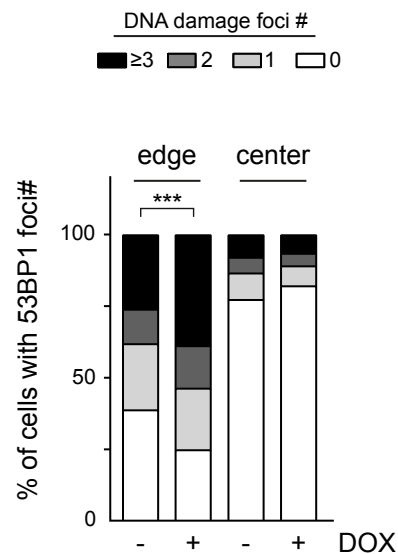

Supplement: Supplementary file 3 [file ACEL-19-e13108-s003.pdf]

A

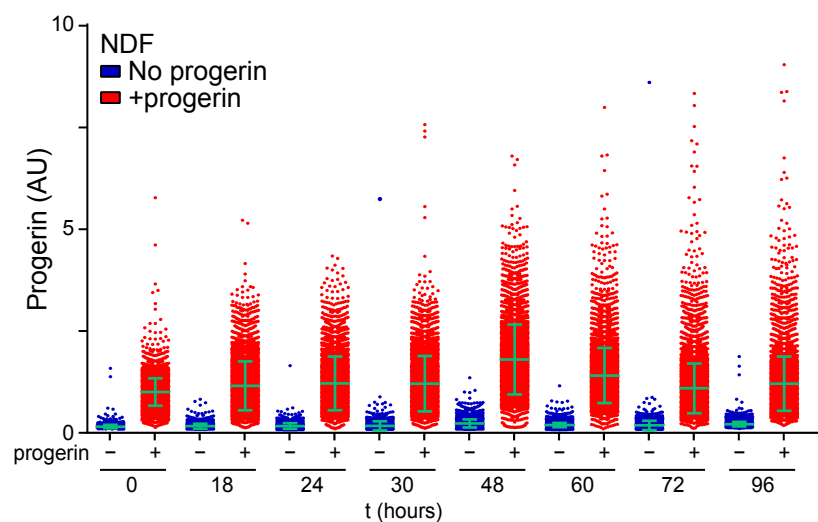

B

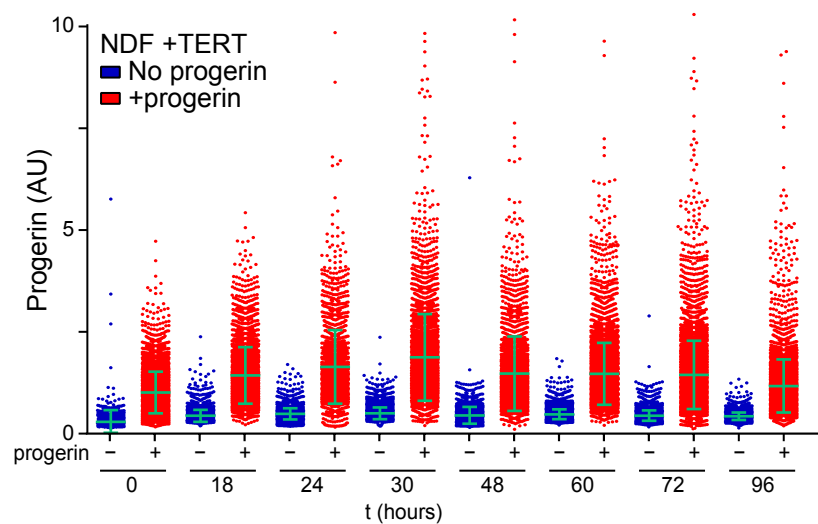

D

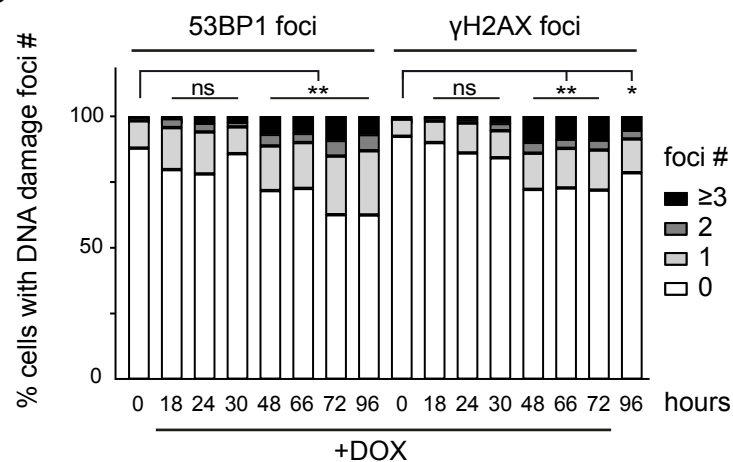

C

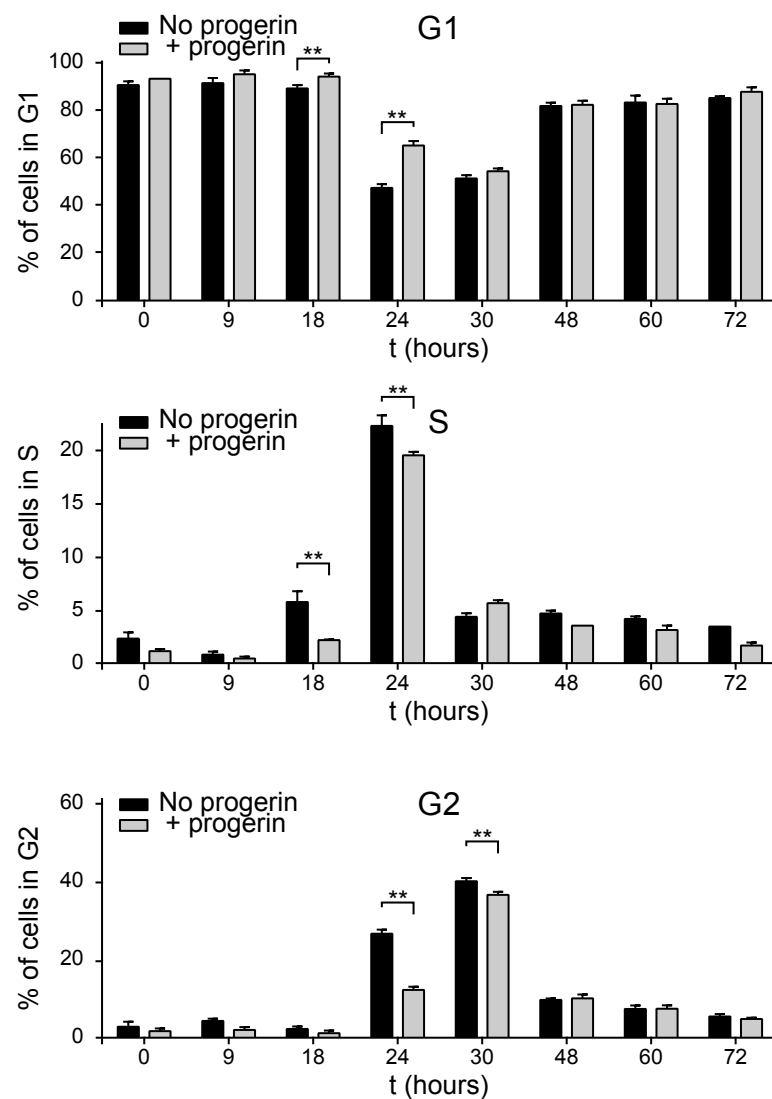

E

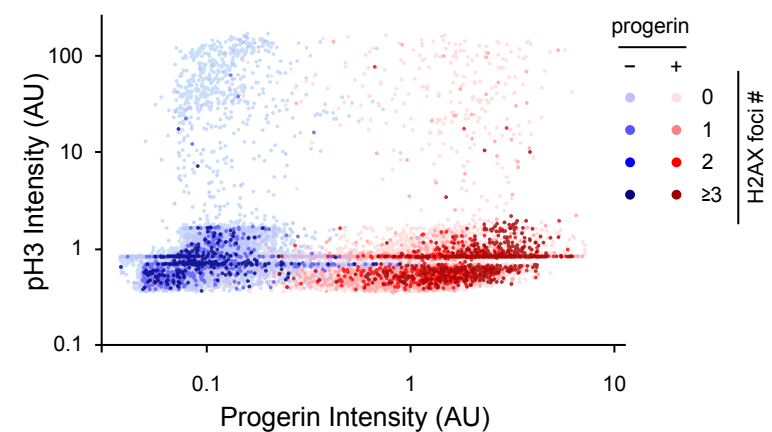

Supplement: Supplementary file 4 [file ACEL-19-e13108-s004.pdf]

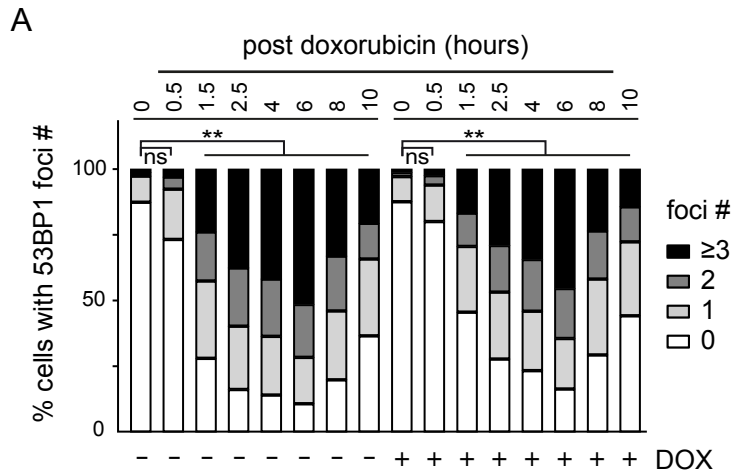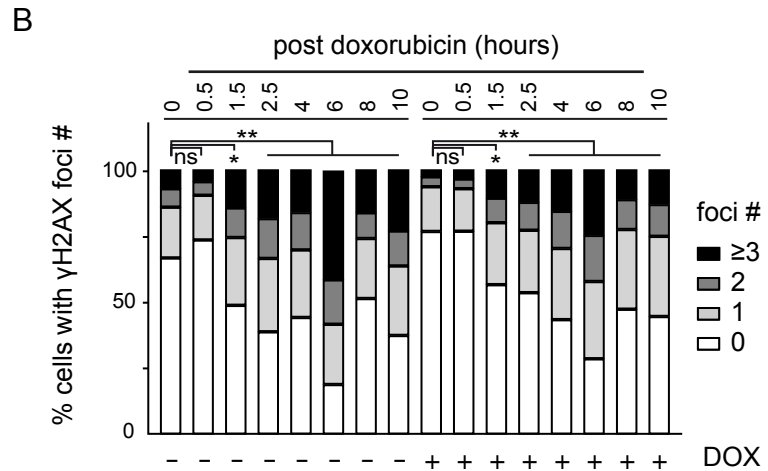

Supplement: Supplementary file 5 [file ACEL-19-e13108-s005.pdf]

A

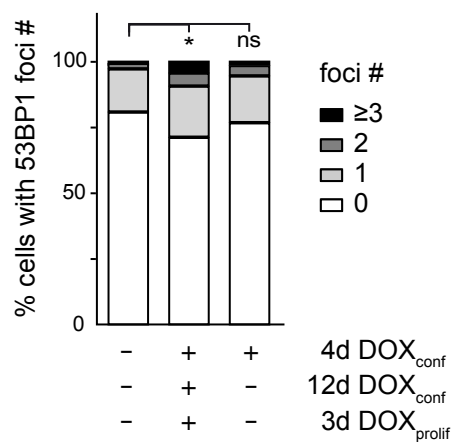

B

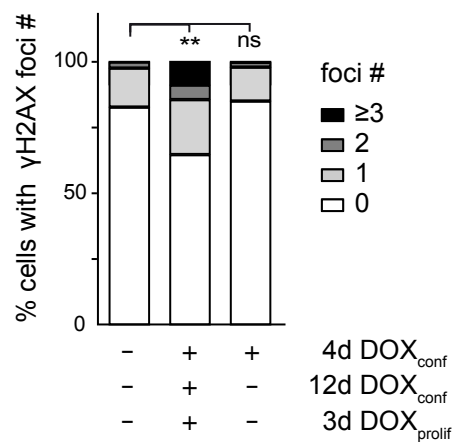

C

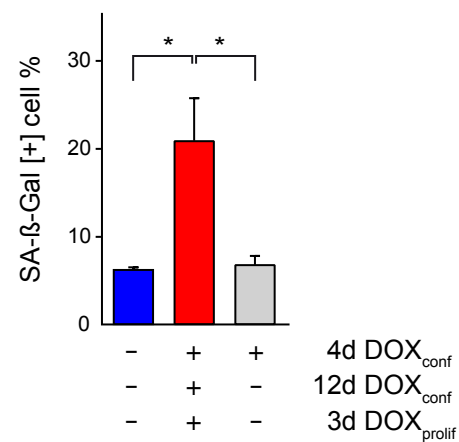

D

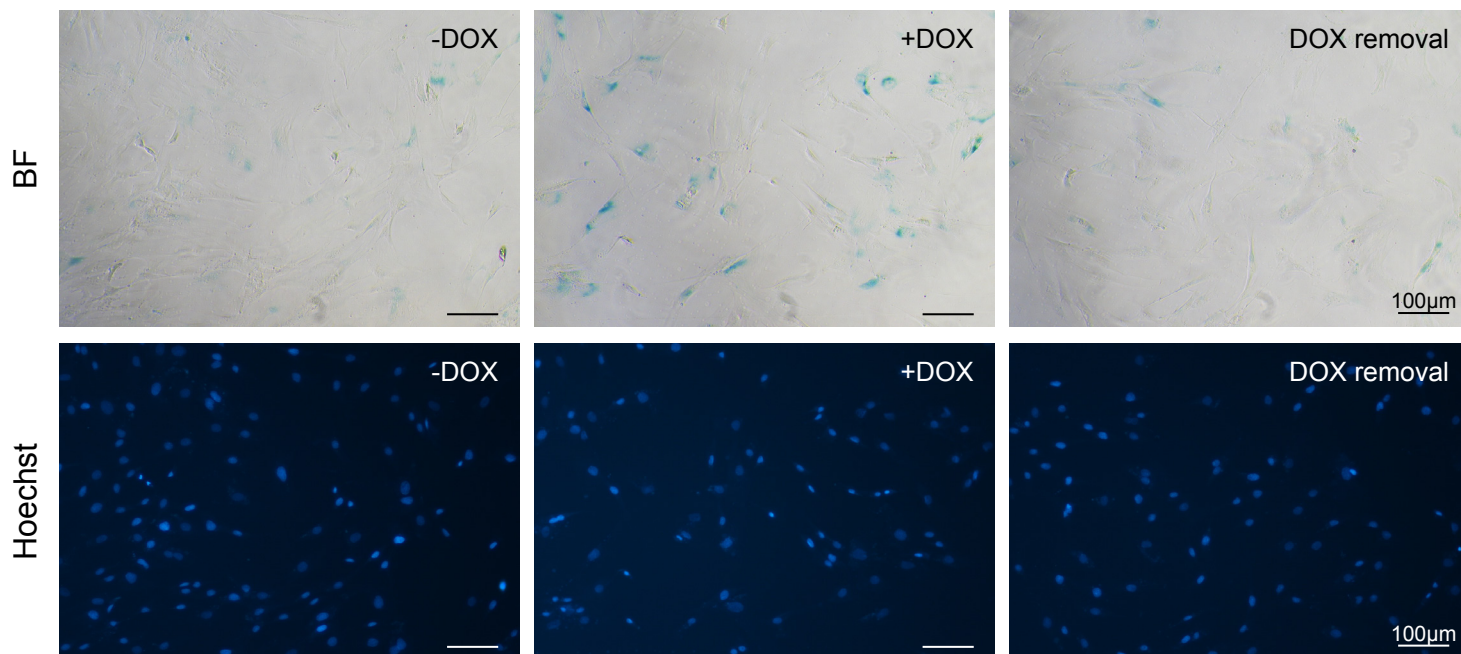

Supplement: Supplementary file 6 [file ACEL-19-e13108-s006.pdf]
